# Breaking the unvirtuous cycle: barriers and opportunities for research and development in Paraguay. A case study

**DOI:** 10.3389/fmed.2023.1266246

**Published:** 2023-11-16

**Authors:** María Lucila Gonzalez Donna, María Luisa Cabañas León, Cinthia Gauna Colas, Alicia Pomata Gunsett, Silvia Ferreira Maniero, David Olivares Osuna, Ezequiel Klimovsky, Lucas Coradini, Diego Enrico, Matías Chacón, Federico Waisberg

**Affiliations:** ^1^Clinical Oncology Unit, Instituto Nacional del Cáncer (INCAN), Ministerio de Salud Pública y Bienestar Social (MSPyBS), Capiatá, Paraguay; ^2^Pathology Unit, Instituto Nacional del Cáncer (INCAN), Ministerio de Salud Pública y Bienestar Social (MSPyBS), Asuncion, Paraguay; ^3^Laboratory Unit, Instituto Nacional del Cáncer (INCAN), Ministerio de Salud Pública y Bienestar Social (MSPyBS), Asuncion, Paraguay; ^4^Lab Manager at Cancer Heterogeneity and Hierarchies Group., Instituto Josep Carreras (IJC), Badalona, Spain; ^5^QUID LATAM Consulting, Buenos Aires, Argentina; ^6^Family Medicine Resident, Hospital Argerich, Buenos Aires, Argentina; ^7^Clinical Oncology, Instituto Alexander Fleming, Buenos Aires, Argentina; ^8^Research Department, Equipo Transdisciplinario de Investigación en Cáncer, Buenos Aires, Argentina

**Keywords:** regulatory science, Paraguay, South America, clinical research, Science Policy

## Abstract

**Introduction:**

Medical research and development (R&D) is an undoubtedly relevant activity to drive innovation, improve healthcare policies and bring patients treatment opportunities for common and rare diseases. Equity and inclusion are matters of concern in research. High-income countries’ research teams are more likely to have more impactful publications, grant funding, and clinical trials than middle or low-income countries. Low budget allocations to R&D and existing gaps in regulatory frameworks are some obstacles to growth. This unvirtuous cycle results in scarce advances in common endemic diseases and the underrepresentation of specific populations in innovative therapeutics research.

**Materials and methods:**

We conducted a policy review and qualitative research to determine the principal characteristics of basic and clinical medical research in Paraguay, as well as barriers and facilitators to improve innovative R&D strategies in this country. To this aim, we examined published articles from 2005 to 2020, the organizational structure of national research agencies, the current regulation framework, and the composition and experience of local research groups and ethical review boards (ERBs). In addition, we performed semi-structured interviews to evaluate perceptions and expectations from different stakeholders, including investigators, ERBs members, sponsor associates, and Regulatory Agency executive staff.

**Results:**

In 2018, Paraguay ranked 10th out of 12 South American countries in total number of publications and cumulative h-index score. Total Gross Domestic Product (GDP) allocation for R&D was 0.15%, ranking eighth out of 12 in the region. In 2021, the number of trials registered on ClinicalTrials.gov was 52, with only 16 ongoing recruiting studies at that time.

Some of the main barriers identified included low incentives for academic careers and lack of experience in pharmaceutical research. An emergent necessity to develop a straight- forward normative framework was detected. Main facilitators included the development of two research initiative programs (PRONII and PROCIENCIA) from CONACYT (National Council of Science and Technology) which were associated with higher budget allocation and total number of publications in the 2011 to 2017 period. A total of six stakeholders participated in the semi-structured surveys. Interviewees highlighted the necessity of a centralized policy to promote R&D, which incorporates investigators and ERBs training, the development of standardized procedures, and the dissemination of research activities. Sponsor associates underlined that real-world evidence may represent a distinctive opportunity to enhance local research.

**Conclusion:**

Coordinated efforts are needed to break the unvirtuous cycle. There is an increasing interest in enhancing health research in Paraguay, materialized in the creation of specific programs that encourage the collaborative work of healthcare providers, basic scientists, and private investors. Nonetheless, a comprehensive approach is needed also to strengthen regulatory agencies and attract external sponsorship. While modern and currently popular topics, including artificial intelligence, real-world data, and translational research may represent key opportunities to seek investment, special policies should be adopted to prioritize research on the determinants of health in the Paraguayan population.

## Introduction

Professional scientific research is considered as an activity of high value and quality. In turn, it is one of the main factors that drive innovation and the economic development of countries. However, research quality and access to funding are not exempt from inequities and are influenced by the socioeconomic situation of each country (1).

The United States and the European Union play a prominent role in this activity, representing around 70% of the registered clinical research studies (2, 3). On the other hand, the research in Latin America reflects a different reality. Some common disadvantageous issues include the low investment in research and development (R&D), the small number of experienced trialists and academic scientists in the region, added to a complex regulatory bureaucracy (4, 5).

Chile, Brazil, and Argentina have led the instrumentation of clinical research in the region (6). Scientific production was remarkably lower in other countries with lower gross domestic product (GDP) in the region, such as Paraguay. Some characteristics of this inequity are likely to be explained by the budget allocation to R&D. Better-paid researchers are more stimulated to have more impactful publications, and grant funding, and develop better research unit areas (4, 7).

Under these circumstances, it is reasonable to expect that an unvirtuous circle is generated in poorer countries. Likely, short-term science policy planning, scarce regional cooperation, and the disarticulation between basic and clinical research constitute factors that hinder breaking this circle (8). Moreover, this unvirtuous cycle may result in scarce advances in common endemic diseases and the underrepresentation of specific populations in innovative therapeutics research.

In this article, we aimed to identify barriers and facilitators for conducting research in Paraguay. By doing so, we expected to establish priorities and evaluate opportunities for improvement to promote R&D in the country.

## Materials and methods

This case study involved two main components: A literature review to analyze the regulatory framework, and the characteristics of past and ongoing research projects, and a semi-structured survey to identify barriers and facilitators to promote clinical investigation in Paraguay.

The first part included the analysis of relevant content from key local institutions and stakeholders. This involved a literature search from the following resources:

1. CONACYT (National Council of Technology and Science) website (9), including the Summary Activity Reports of 2017 and 2018 (10, 11).
2. Publications of researchers with current affiliation in Paraguay were analyzed using Scimago and Scopus databases. SJR (SCImago Journal Rank) and Impact Factor scores were collected. We accessed ClinicalTrials.gov, FDA (Food and Drug Administration), and Pubmed websites to evaluate past and current clinical research from Paraguayan authors, including publications from 2011 to 2021. Research activity was compared with other South American countries.
3. The current DINAVISA (Dirección Nacional de Vigilancia Sanitaria is the National Health Regulation Department regulatory framework was explored using institutional websites, and available publications from international agencies, such as the EAMI (Red de Autoridades de Medicamentos de Iberoamérica; Iberoamerican National Health Autority Network) (12). DINAVISA activity was evaluated using the assessment of National Regulatory Authorities (NRA) for Medicines guidelines of the WHO/PAHO (World Health Organization / Pan American Health Organizations) (13).
4. The list of registered ERBs (Ethics Review Boards) was obtained from the Health Ministry webpage (14). A digital survey was conducted to collect information on the identified ERBs. UNESCO (United Nations Educational, Scientific and Cultural Organization) guidelines for surveys were followed (15). Potential respondents were identified based on existing Paraguayan authors’ publications and recommendations of opinion leaders. The information asked included the date of creation, the current number of members, the existence of standardized operational procedures documents, meeting cadence, and the number of evaluated cases during the previous year.
5. Research sponsors were identified using the 2014 Paraguayan Pharmaceutical Profile Report available on the PAHO website (16, 17).

In the second part of this study, a semi-structured interview was performed with relevant stakeholders in Clinical Investigation activities. The interview candidates were selected after consultation with regional and national opinion leaders, taking into account their expertise, number of publications, and past or current interest in conducting research in Paraguay. Interviewees were asked about their research experience, barriers and facilitators experienced in the past, and what opportunities should be prioritized to develop research in the country.

The following professionals accepted a one-hour interview:

- A DINAVISA associate.
- A Paraguayan researcher with current activity in Paraguay.
- A Paraguayan researcher with current activity in Spain.
- 2 CROs (Contract Research Organizations) associates from Latin America.
- A Medical Director of an International Pharmaceutical company.

For the quantitative analysis, data were summarized using medians and interquartile ranges. The interviews were recorded and transcribed for data extraction. Additional questions were made by e-mail, when necessary. All the literature review was conducted from September 2020 to June 2021. Qualitative research was performed in July 2021.

The protocol of this study was approved by the ERB of the National Institute of Cancer of Paraguay (INCAN, 7th of February of 2020).

## Results

### Literature review

The research framework in Paraguay is complex and different stakeholders were identified in our review. In the following sections, we will describe the characteristics of current programs that promote research in this country, the impact of scientific production, the demographics of ERBs staff in Paraguay, and the regulatory framework analysis by WHO/PAHO guidelines. Finally, we will summarize the results of the semi-structured interviews that were conducted with the included stakeholders.

#### National research career agency

The CONACYT (Consejo Nacional de Ciencia y Tecnología; National Council of Science and Technology) is a government entity, which oversees scientific policy planning. It promotes academic and private research through three main programs. PRONII (Programa Nacional de Incentivo al Investigador) is the National Program for Researcher Career Support. This program was initiated in 2011 to promote the professionalization of the scientific career in Paraguay. There are four career categories in the program, including research candidates, and Level I to III staff researchers. In 2020 more than 50% of the researchers included in PRONII were categorized as research candidates, and only 4% were defined as level III investigators. In this program, a total of 249 researchers were associated with Medical Science and Health disciplines. 67 (27%) were physicians, including 33 Level I, four Level II, and only one Level III researcher. Only 10 of this group had pharmacological research background, and 6 of them reported having executed the majority of their research activities in other countries.

PROINNOVA (Programa de Innovación en Empresas Paraguayas) is the Innovation Program for Paraguayan Companies. It was approved in 2017 and financed by the Inter-American Development Bank. This program was intended to promote sustainable research with profit-oriented goals. During that year, 18 companies were co-financed. None of these projects were dedicated to the healthcare area.

PROCIENCIA (Programa Paraguayo para el Desarrollo de la Ciencia y la Tecnología) is the Paraguayan Program for the Development of Science and Technology It was created in 2014. This program regularly provides grants for the scientific community in Paraguay. A maximum of 90,000 USD is yearly assigned for each winning research project.

In 2017, the investment in research, development, and innovation in Paraguay was 0.20% of the Gross Domestic Product (GDP), ranking eighth out of 12 in the South American region (Table 1) (5). This budget had been increasing gradually since 2011. In that year, the investment in Paraguay was lower than its neighboring countries, including Argentina (0.53%) and Brazil (1.28%), and the average GDP invested in Science in Latin America and the Caribbean (0.67%). 77.4% of the funds were concentrated in the public sector, of which 31.9% were financed by the CONACYT programs. Only 0.2% of the total research funding was financed by private stakeholders. Medical and Health Sciences was the second most subsidized area, right after Agricultural and Veterinary Sciences.

**Table 1:** Indexed publications and gross domestic product investment in South American countries.

Country	Population*	Per-capita GDP (USD)*	Investment in R&D (% of gross GDP)^#^	h-index in 2020
Brazil	210.147.125	16.199	1.27	530
Argentina	48.258.494	20.161	0.53	393
Chile	28.067.000	27.058	0.36	349
Colombia	44.938.712	17.406	0.24	261
Peru	33.105.273	13.993	0.12	212
Venezuela	19.107.216	10.968	0.12	205
Uruguay	3.529.014	24.453	0.41	179
Ecuador	17.300.000	11.732	0.44	149
Bolivia	11.383.094	7.943	0.16	119
Paraguay	7.152.014	13.471	0.15	82
Guyane	761.00	8.524	NA	41
Suriname	524.00	14.497	NA	38

GDP, Gross Domestic Product; R&D, Research and development; NA, Not available. *Incorporated information was based on 2018 indicators. ^#^Investment was calculated incorporating data retrieved between 2008 and 2018 using World Bank indicators (5).

**Figure 1:**
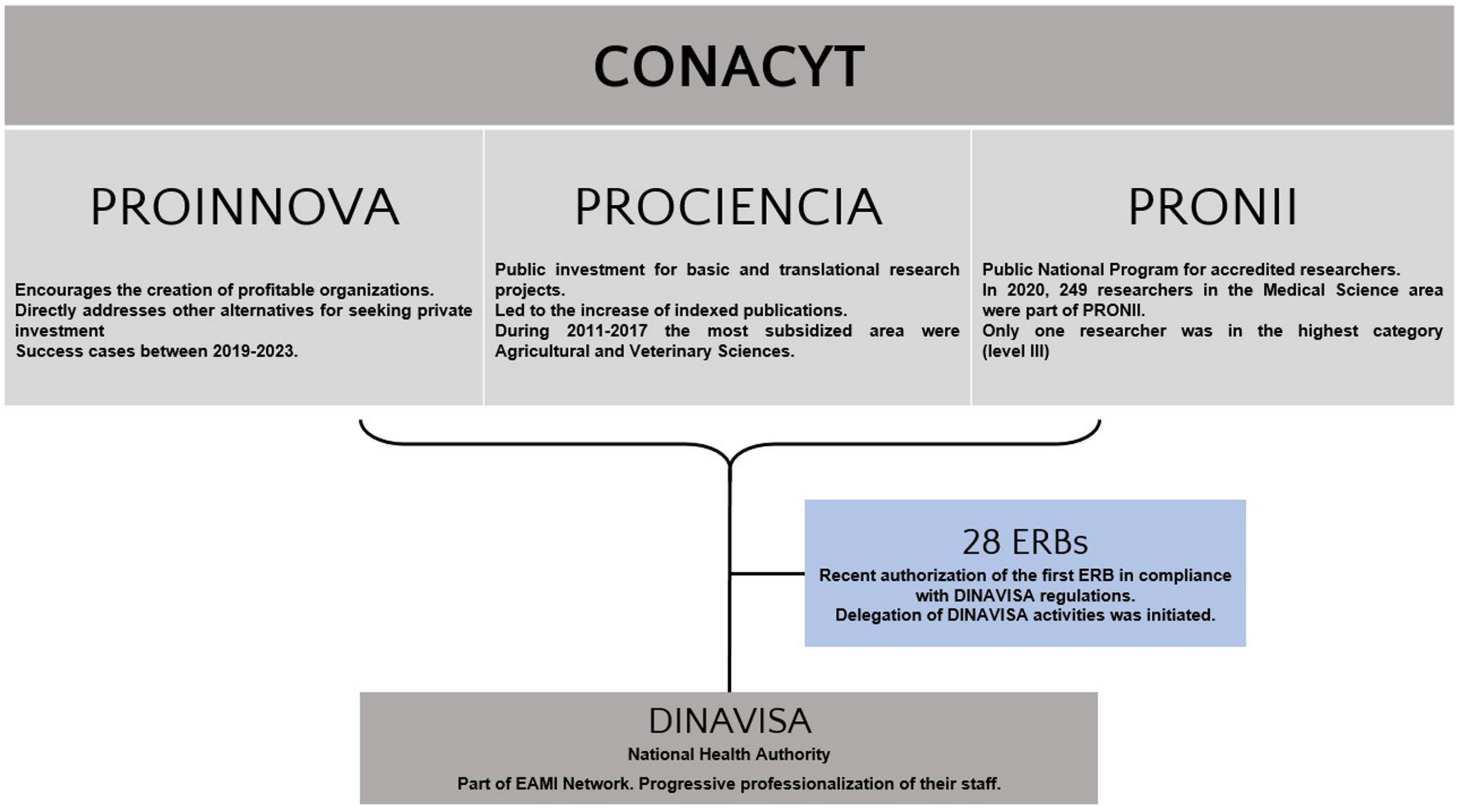
Summary of Paraguayan institutions involved in research evaluation and scientific policy planning. PROINNOVA, Programa de Innovación en Empresas Paraguayas; Paraguayan Companies Innovation Program; PROCIENCIA, Programa Paraguayo para el Desarrollo de la Ciencia y la Tecnología; Paraguayan Program for Science and Technology Development; PRONII, Programa Nacional de Incentivo al Investigador; National Program for Research Support; ERB, Ethical Review Board; DINAVISA, Dirección Nacional de Vigilancia Sanitaria; National Health Regulation Department; EAMI, Red de Autoridades de Medicamentos de Iberoamérica; Iberoamerican National Health Autority Network.

Figure 1 summarizes the central programs of the National Research Career Agency.

#### Demographics of research activity of physicians in Paraguay

Up to 2018, 35 (52.2%) of the 67 physician researchers did not have Pubmed-indexed publications. 31 (46.2%) appeared in less than 10 publications, and only one physician was listed in more than 20 publications.

Considering the Scimago Quality Index, 24% of this group reported publications in journals without an Impact Factor score. The cumulative Impact Factor was less than 20 for 41 (61%) researchers, and only one physician had a cumulative impact factor greater than 100.

Since the creation of PROCIENCIA, in 2014, the number of publications of Paraguayan researchers, indexed in Scopus and Web of Science, has increased. From 2014 to 2017, the total number of publications was 164, 226, 255, and 329, for the respective years of the period.

According to ClinicalTrials.gov, the total number of Clinical Trials with registered Paraguayan investigators from 2005 to 2021 was 52, with only 16 recruiting studies during that year. From this list, 19 clinical trials investigated drugs or therapies, and 15 were funded by private sponsors. Considering the latter, a total of 5, 11, and 3 were Phase 2, 3, and 4 clinical trials, respectively. The participating sites mostly were public hospitals, and one private center specialized in hematological malignancies.

#### Ethical review boards in Paraguay

A digital survey was conducted to retrieve information regarding existing ERBs in Paraguay. A total of 161 potential interviewees, including health, scientific and educational institutions, associated with research activities were identified within the country. 128 respondents completed the survey, 73 from healthcare services (65 public and 8 private), 28 from universities (11 public, 17 private), 20 from scientific societies, and four from clinical research sites.

Through this survey, a total of 28 ERBs were individualized. 17 (61%) were based on universities, 6 (21%) on public healthcare institutions, 2 on scientific societies and the remaining on private institutions, including a healthcare center, an academic institution, and an external ERB associated with a research site. 18 (68%) ERBs declared to be specialized in research activities.

In 11 cases, the constitution was after 2013. 20 (71.8%) of them communicated that they had standard operative procedures, and 19 (67.8%) maintained meeting recordings. When asked, most of the analyzed ERBs (*n* = 15, 47%) specified that they did not schedule regular meetings and that sessions were held based on necessity.

ERBs composition reflected multidisciplinary research in only four cases. In the remaining cases, their staff did not include lawyers, social scientists, or community members.

Table 2 reflects the characteristics of the ERBs identified through the survey. As detailed, most of the included ERBs analyzed less than 50 research projects during the period from 2015 to 2020.

**Table 2:** ERBs activity characteristics in the 2015–2020 period.

Type of institution	University (*n* = 16)	Hospital-based (*n* = 4)	Private research institution (*n* = 1)
Number of analyzed submissions in 2020			
1–50	12	1	0
51–100	0	0	0
>100	2	1	0
No data	2	2	1
Number of analyzed submissions between 2015 and 2020			
1–50	8	0	0
51–100	2	1	1
>100	2	2	0
No data	4	1	0

#### Regulatory framework and implications for clinical trials

The National Regulatory Authority (NRA) in Paraguay is DINAVISA, which is overseen by the National Health Ministry. It was created in 1997, and it is recognized in the PAHO list of regional regulatory authorities, DINAVISA has been incorporated into the EAMI network which aggregates 22 NRAs from Latin America, Andorra, and Portugal.

Up to 2020, there were no further registered regulations approved by DINAVISA to organize research activities since its creation. According to available reports, applicable legislation includes South Common Market (MERCOSUR, due to its Spanish abbreviation) Resolution 129/96, the International Council for Harmonization of Technical Requirements for Pharmaceuticals for Human Use (ICH), Council for International Organizations of Medical Sciences (CIOMS) Ethical Guidelines, and the Helsinki Declaration (18).

DINAVISA approved the first clinical trial in Paraguay in 2008, and a total of five site inspections were conducted between 2008 and 2011. No disciplinary sanctions were issued since the initiation of its activity.

Table 3 reflects the application of PAHO criteria to available regulations and procedures of DINAVISA. Considering retrieved information, only one of the requirements of the PAHO criteria list was verified by the Paraguayan NRA.

**Table 3:** Current characteristics of DINAVISA in 2020, according to PAHO criteria.

Category	Requirement	Priorities	Fulfillment
Ethics framework	Standardized procedures to evaluate Clinical Trials Conflict of Interests Policies Code of conduct for ERB staff	Critical	No
	Detail of methodology used for Clinical Trial evaluation	Necessary	No
Structure	Central organization of Ethics Advisory Boards activities	Necessary	Yes
Evaluation procedures	Standard procedures to evaluate Clinical Trial Protocols and Amendments Procedures for evaluation of other research documentation, including Investigator Manual, Research Plan and Data Collection procedures Standard procedures regarding the decision processes of NRA Regular auditories performed to research sites Maximum time intervals for the NRA to finish the evaluation of a clinical trial submission	Critical	No
	Standardized procedures for the Informed Consent Form process Written authorizations and approval reports are delivered to sponsor delegates	Necessary	No
Registry of NRA activities and information availability	Database of approved and rejected clinical trials File of all clinical trials, including amendments, exemption and evaluation reports Availability of databases of approved and rejected clinical trials	Critical/ Necessary	No

NRA, National Regulatory Authority; ERB, Ethics Review Board.

#### Private investment in clinical research

The National Group of Pharmaceutical Companies in Paraguay (CIFARMA) reported that in 2018, a total amount of USD 100 million was invested in the country, which represented an annual increase of 10%. During that year, a soft capsule and a pharmaceutical biotechnology company were opened in Paraguay.

Nonetheless, only one international pharmaceutical company is directly based in the country, which limits clinical research activity in Paraguay.

### Semi-structured interviews with research stakeholders

A total of six stakeholders accepted a one-hour interview. The questions addressed expectations, barriers, and facilitators regarding clinical research in Paraguay. All the interviews were conducted by the same evaluator and were performed in a private environment.

Firstly, the DINAVISA associate expressed that they were willing to enhance clinical research in Paraguay. Some of the difficulties experienced included the lack of adequate training. National support, including the hiring of more administrative staff time, was identified as the main need to be addressed. On the other hand, the DINAVISA associate underlined that the number of clinical trial submissions increased during 2018 and 2019. He expected that this would lead to the creation of new documentation and guidelines. The delegation of DINAVISA activities to ERBs was mentioned as a strategic step to promote research in a country with economic constraints.

Two investigators, with previous research experience in Paraguay, were interviewed: Researcher A was a Clinical Investigator based in Paraguay, and Researcher B is currently working in a European center.

Researcher A described that his site was prepared to conduct phase II and III clinical trials and that a total of 26 employees were trained to perform clinical trial activities. Patient recruitment activities had been adequate. In his view, the principal barriers were the lack of specific regulations for trial supplies importation. Additionally, he underlined that healthcare coverage may differ across the different Paraguayan sub-systems and that this might discourage the conduction of studies that require a long-term follow-up. The researcher claimed that “Sponsors are interested in Paraguay for early drug development clinical trials.” He considered that the next step was to disseminate and train more physicians and coordinators in clinical trial procedures.

Researcher B experienced difficulties in pursuing an experimental trial that would assign 40 cancer patients to a drug associated with their tumor molecular profile. He mentioned that he was unable to finish the research due to a governmental budget re-assignation. Researcher B also stated that some of the actual barriers also included the high workload of healthcare providers, pending regulations on logistics, and the lack of experience with the DINAVISA. Nonetheless, researcher B has praised the activity of the ERBs, explaining that in his experience, the time to clinical trial approval was between 6 to 8 weeks.

Three sponsor associates were interviewed: Associate A, from an international pharmaceutical company based in Argentina, and associates B and C, who worked in regional CROs.

Associate A described pathways that should be considered by DINAVISA to accelerate clinical research development. He stated that “DINAVISA needs to offer speed and predictability, and this is a first step to consider a site for its selection.” Sites also need to ensure protected time for all the involved staff for research activities. Other essential activities that are regularly taken into evaluation include regional start-up times and disease prevalence. In the interviewee’s perspective, the delegation of the evaluation of clinical trial protocols to central ERBs, and the adoption of importation regulations were the most important steps to take. He also addressed that the adoption of new technologies, and the collection of real-world evidence may represent important opportunities to gain academic research experience.

Associate B highlighted that compliance with recruitment expectations and short start-up times are the central factors to select a research site. For the Paraguayan case, he also remarked that importation logistics should be improved to accelerate start-up times. He mentioned contacting Paraguayan researchers to evaluate clinical trial conduction feasibility but explained that they were not interested in opening investigation sites.

Associate C’s company conducted clinical research in Paraguay and concluded that it was a valuable experience. He recommended that the focus should be to improve local regulations and decrease start-up times. He also strengthened the need for training local monitors. He remarked that CROs were experiencing huge difficulties to hire, train and retain associates.

## Discussion

This case study was a comprehensive effort to understand the characteristics of clinical research in this developing South American country. To evaluate how research activities are organized and developed in the country, we will summarize our findings using a “Strengths, Weaknesses, Opportunities, and Threats (SWOT)” analysis.

The strengths of the Paraguayan research organization include its international recognition, a willingness to interact and promote clinical research, the increasing budget invested in science, and the recent adoption of policies that promote science-related business development. The increasing trend of peer-reviewed publications reflects an adequate response to the adopted funding strategy.

Some common weaknesses identified included the lack of specific NRA regulations to accelerate the clinical research start-up process, such as the importation of necessary supplies. An essential requirement is to better assess the training needs of all the involved participants, including DINAVISA and ERBs staff, and clinical investigators. The scarce funding and the small number of experienced staff in academic research are also key areas that should be prioritized to promote studies not associated with pharmaceutical interests, such as community-based participatory research.

Specific endemic diseases, such as tuberculosis, yellow fever, and penile cancer are particularly prevalent in Paraguay, and efforts to promote impactful research should underscore these relevant topics for global health (19–21). Understanding the social determinants of health in this population, and collecting epidemiological data is crucial to promote implementation programs to prevent, diagnose, and treat these complex regional diseases. Other opportunities especially targeted for pharmaceutical research include low tax rates and relative macroeconomic stability.

The main threat to confront is the lack of a long-term science program to guarantee the continuity of the implemented strategies. Political alternation and the absence of integrated regional policies to promote research might hamper the development of academic and clinical research in Paraguay.

The COVID-19 pandemic represented a unique opportunity to promote research in Paraguay. Since 2021, five clinical trials that investigated the role of COVID-19 treatment or vaccines incorporated Paraguayan research sites. The Paraguayan National Cancer Institute (INCAN) created the first ERBs formally accredited by DINAVISA in September 2022. Additionally, during that year, DINAVISA conducted an audit of a research site for a COVID-19 vaccine study. Additionally, different training sessions were held to train members from 5 ERBs and NRA agents (22, 23).

Although these opportunities, often associated with private funding, represent important steps to gain experience, it is essential to maintain a “90/10” perspective at the moment of defining science policy (24). The research with better chances of impacting the Paraguayan population will be probably represented by studies that assess “unfancy” local or regional problems and will be most likely conducted by academic investigators. Under this perspective, it is critical to incorporate academic and clinical researchers to discuss knowledge transfer strategies and the prioritization of topics for national funding. For instance, in Argentina a government-based integrative network was developed to stimulate conferences and funding opportunities for translational research (25). Other strategies might be incorporated, such as integrating a committee that includes basic and clinical investigators and defining prioritized research lines for CONACYT grant programs.

Ciocca and Delgado have described problems that Argentinean academic researchers commonly face (7). In addition to the low amount of budget assigned to academic research in South America, the authors emphasized other topics of concern such as the obstacles to supplies importation, the lack of transparency in the funding assignation process, the extremely bureaucratic requirements needed for career development, and the existence of more profitable opportunities out of country (“brain drain”).

Lessons are to be learned. Some common regional issues support the necessity of harmonizing science policy agendas in South America. Modern technology research, such as translational research, artificial intelligence, the growing demand for global epidemiological data, and the need to focus on social determinants of health, represent strategic areas that may be the basis for regional cooperation. Not surprisingly, MERCOSUR promoted grant opportunities for “Artificial Intelligence” and “Assistive Technology” in 2020 and 2021, respectively (26, 27).

It can be concluded that the existence of an unvirtuous circle is a key aspect of developing countries. Suboptimal investment and the lack of clear regulatory frameworks lead to low scientific production, a scarce number of trained academic investigators and collaborators, and inadequate structures to conduct research. Under these circumstances, it might be expected that private investment will remain deficient. Nonetheless, this circular reasoning is breakable.

**Figure 2:**
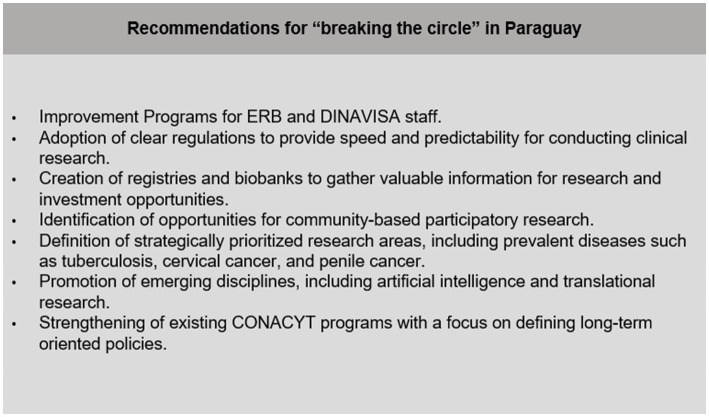
List of recommendations for “breaking” the unvirtuous circle. ERB, Ethics Review Board; DINAVISA, Dirección Nacional de Vigilancia Sanitaria; National Health Regulation Department; CONACYT, Consejo Nacional de Ciencia y Tecnología; National Council of Science and Technology.

Central organization and regional cooperation are essential to address most of the weaknesses and threats identified in this report, including the lack of articulation between public and private research areas, the sparse number of experimented trialists, and the necessity of clearer regulations to ensure transparency and to accelerate the start-up process of clinical trials. We consider that the important steps were taken in the last few years. However, all the involved participants, including decision-makers, physicians, sponsors, and regulatory agents are strongly needed to combine efforts toward a cultural change in how we think about science policy-making, prioritizing long-term goals and regional cooperative research. A summary of the main recommendations obtained from the literature and policy reviews, and the semi-structured survey is represented in Figure 2.

The limitations of this study should be considered. Our literature review was not systematic, and consequently, some of our findings are prone to bias. Our review does not reflect most of the changes that were incorporated after the COVID-19 pandemic, which may hamper the current applicability of some of our results. Finally, only limited stakeholders were incorporated into the semi-structured review, which may also interfere with the generalization of our analysis. Further studies should also incorporate the appreciation of other relevant actors, including ERB members and CONACYT directives.

## Conclusion

Several barriers were identified for conducting research in Paraguay. The necessity of improving the NRA regulatory framework, and the training of ERBs members and researchers are some of the priorities to be addressed to break the unvirtuous circle. Importantly, the promotion of transparent policies and the definition of long-term objectives, including the articulation of basic and clinical research, and the allocation of budget for both profitable and academic research are essential to foster a scientific production that responds to the population’s needs.

## Data availability statement

The datasets presented in this article are not readily available because the original data was based on interviews, that were recorded but they are not available for sharing. Requests to access the datasets should be directed to fwaisberg@alexanderfleming.og.

## Ethics statement

The studies involving humans were approved by Ethical Research Board of Instituto Nacional del Cancer (INCAN) of Capiatá, Paraguay (National Cancer Institute). The studies were conducted in accordance with the local legislation and institutional requirements. The participants provided their written informed consent to participate in this study.

## Author contributions

MD: Formal analysis¸ Funding acquisition, Project administration, Resources, Supervision, Validation, Visualization, Writing – original draft, Writing – review & editing, Conceptualization, Data curation, Investigation, Methodology, Software. ML: Investigation, Methodology, Writing – original draft. CC: Investigation, Methodology, Writing – original draft. AG: Investigation, Methodology, Writing – original draft. SM: Investigation, Methodology, Writing – original draft. DO: Investigation, Methodology, Writing – original draft. EK: Conceptualization, Investigation, Project administration, Supervision, Writing – review & editing. LC: Conceptualization, Data curation, Investigation, Methodology, Writing – original draft, Writing – review & editing. DE: Investigation, Methodology, Writing – review & editing. MC: Investigation, Methodology, Validation, Writing – review & editing. FW: Formal analysis¸ Funding acquisition, Project administration, Resources, Supervision, Validation, Visualization, Writing – original draft, Writing – review & editing.
